# Clinical scientists’ early career choices and progression: an exploratory mixed methods study

**DOI:** 10.1186/s12913-021-07064-1

**Published:** 2021-10-06

**Authors:** Megan Smith, Jaimini Patel, Sandie Gay, Ian Davison, Sharon Buckley

**Affiliations:** 1 Formerly Birmingham Medical School, University of Birmingham, Birmingham, UK; 2 Formerly National School of Healthcare Science, Health Education England, Birmingham, UK; 3 Formerly School of Education, University of Birmingham, Birmingham, UK; 4 Birmingham Medical School, University of Birmingham, Vincent Drive, Edgbaston, B15 2TT Birmingham, United Kingdom

**Keywords:** Clinical scientists, Career choice, Progression, Job embeddedness, Mixed methods, Workforce retention

## Abstract

**Background:**

Understanding the influences on healthcare professionals’ career choices and progression can inform interventions to improve workforce retention. Retention of health professionals is a high priority worldwide, in order to maintain expertise and meet the needs of national populations. In the UK, investment in clinical scientists’ pre-registration education is high and the need to retain motivated scientists recognised.

**Methods:**

We conducted a mixed methods study to investigate the career choices and progression of early career clinical scientists. First job sector and salary of trainees who completed the UK pre-registration Scientist Training Programme (STP) between 2014 and 2019 were analysed using descriptive statistics and Chi-Squared tests. Semi-structured interviews conducted with volunteer practising clinical scientists who completed the programme in 2015 or 2016 were analysed thematically and reviewed for alignment with theories for understanding career choice and workforce retention.

**Results:**

Most scientists who completed the STP between 2014 and 2019 obtained a post in the UK National Health Service (NHS) and achieved the expected starting salary. Life scientists were more likely to work in non-NHS healthcare settings than other scientific divisions; and physiological scientists less likely to achieve the expected starting salary. Experiences during training influenced career choice and progression 0–3 years post qualification, as did level of integration of training places with workforce planning. Specialty norms, staff turnover, organisational uncertainty and geographical preferences influenced choices in both the short (0–3 years) and longer term (5 + years). Interviewees reported a strong commitment to public service; and some could foresee that these priorities would influence future decisions about applying for management positions. These factors aligned with the components of job embeddedness theory, particularly that of ‘fit’.

**Conclusions:**

Training experiences, personal values, specialty norms and organisational factors all influence UK clinical scientists’ early career choices and progression. Job embeddedness theory provides a useful lens through which to explore career choice and progression; and suggests types of intervention that can enhance the careers of this essential group. Interventions need to take account of variations between different scientific specialties.

**Supplementary Information:**

The online version contains supplementary material available at 10.1186/s12913-021-07064-1.

## Background

High quality patient care depends in large part on the expertise and morale of the health workforce [1]; and health services in many countries, including the UK, find it difficult to recruit and retain motivated professionals to meet the needs of their populations [2, 3].

Understanding the influences on health professionals’ career choice and progression can inform efforts to improve retention [4]. However, despite the need to retain motivated and enthusiastic individuals in many health professions, research into the factors influencing career choices has, to date, focussed mainly on medicine [5–7]. Furthermore, whilst various theories have been used to aid understanding, including Bronfenbrenner’s socio ecological model of human development [8, 9] and Mitchell’s job embeddedness theory [10–12], little is known about the extent to which insights from these theories might apply to other health professional groups.

In the UK, as in other countries, clinical scientists make up a significant proportion of the healthcare science workforce [13]. Healthcare scientists are essential for clinical diagnosis and decision-making; and have been central to the national response to COVID-19 [14]. Recruitment and retention, including that of healthcare scientists, are priorities for the UK National Health Service [3, 15, 16]. We have investigated the career choice and progression of early career clinical scientists in the UK, with a view to helping educators and workforce managers design interventions that improve recruitment and retention of this necessary healthcare workforce.

The UK Scientist Training Programme (STP) is a three-year post-graduate pre-registration programme for aspiring clinical scientists. It involves a remunerated training post blended with a Master’s degree in clinical science. Trainees specialise in one of 30 specialties within four scientific divisions: life sciences, physical sciences, physiological sciences and bioinformatics [17]. All trainees begin their training on the NHS Agenda for Change (AfC) salary Band 6 [18]. Upon successful completion of the STP, their expected NHS salary is Band 7. All completers are eligible to register as a clinical scientist with the profession’s regulator, the Health and Care Professions Council [19].

Our exploratory study has sought to answer the following questions: how have the careers of early career clinical scientists developed since they completed the STP; how do their career destinations so far compare with their aspirations; what factors have influenced their career choices to date; and how do they see their career developing over the next 5 years?

## Methods

 For our mixed-method exploratory investigation (University of Birmingham Research Ethics Committee approval number ERN_18–0107), participants were early career clinical scientists who completed the Scientist Training Programme (STP) between 2014 and 2019.

Data from the annual STP exit surveys for 2014–2019 were analysed to identify newly qualified scientists’ first job sector and salary. Chi-squared tests (SPSS Statistics version 24 (IBM)) were used to test whether associations between variables were significant: p-values < 0.05 were considered significant.

Semi-structured interviews were held between June and December 2018. The National School of Healthcare Science (NSHCS) invited individuals who completed the STP in 2015 and 2016 and therefore had 2–3 years early career experience to volunteer for the study via email. To reduce the risk of identification, only individuals from specialties with more than eight trainees a year were invited. Volunteers registered their interest by emailing the contact address of the research group. Interview questions explored scientists’ career choice and progression to date and their future career plans (see Additional file 1).

Interviews were recorded, transcribed intelligence verbatim (i.e. ‘filler’ words e.g. ‘erm’ and repeated words or phrases were removed) and analysed thematically, following Braun and Clarke’s six stages [20]. Transcripts were coded independently by two researchers using NVivo version 12 [21]. Analysis was inductive and semantic as opposed to deductive and latent [20]. The two researchers discussed emergent codes and patterns throughout the coding phase allowing comparison and standardisation of codes. Once coding was complete, codes were organised into themes.

Themes were reviewed for alignment with Bronfenbrenner’s socio-ecological model of human development [8] and with job embeddedness theory [10]. Bronfenbrenner classifies factors affecting human development into ‘systems’ ranging from the ‘micro’ to the ‘macro’ level; whilst job embeddedness theory uses the concepts of ‘link’, ‘fit’ and ‘sacrifice’. These two frameworks were chosen as most appropriate for our study after reviewing the existing literature on health professionals’ career decision-making. Both are well-articulated frameworks and both have been used to aid understanding of the influences on the career choices of doctors [10, 11], a health profession which, like the clinical sciences, is scientific and specialty-based.

## Results

### Immediate career destinations of STP completers

Between 2014 and 2019, 88 % (*n* = 1226) of completing trainees returned the STP exit survey.

**Fig. 1:**
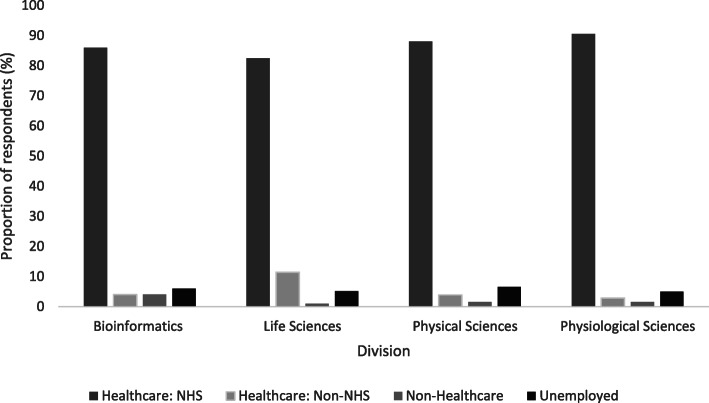
First job sector destinations of STP completers by scientific division; bioinformatics (*n* = 50), life sciences (*n* = 410), physical sciences (*n* = 385), physiological sciences (*n* = 381). Note: Healthcare: NHS includes related public sector bodies such as Public Health England. Healthcare: non-NHS includes private healthcare and other scientific organisations

Most survey respondents (86 %) reported first post STP employment with the UK NHS (Fig. 1). Job sector of first employment was significantly associated with division (X^2^(9, *n* = 1226) = 35, *p* < .001). This appears to be due to variation in the percentage of completers staying in or leaving the NHS; 82 % of life scientists stayed compared to 90 % of physiological scientists. More than a tenth (11 %) of those from life sciences gained employment in a non-NHS healthcare setting, compared to 4 % from bioinformatics, 4 % from physical science and 3 % from physiological sciences.

### Starting salaries of STP completers

Most completers whose first post was in the NHS reported receiving the expected Band 7 salary (67 %), whilst a third (31 %) remained on Band 6. Salaries for those whose first post was outside the NHS were more variable, with 20 % of those entering non-NHS healthcare settings receiving a Band 8 equivalent and 9 % reporting a decrease in salary.

**Fig. 2:**
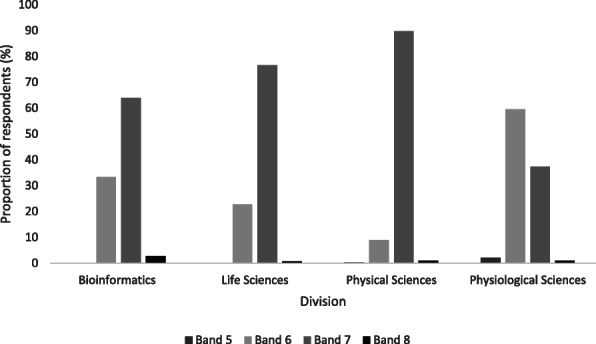
Starting salaries of STP completers entering the NHS by scientific division. The proportion of STP completers from each division entering the NHS is shown, with starting salaries as AfC Bands 5–8. Respondents were from bioinformatics (*n* = 36), life sciences (*n* = 303), physical sciences (*n* = 312), physiological sciences (*n* = 329)

NHS salary on completion was significantly associated with scientific division (X^2^(9, *n* = 980) = 225, *p* < .001). This appears to be primarily due to lower starting salaries in the physiological sciences: 64 %, 77 % and 90 % of respondents from bioinformatics, life and physical sciences respectively commanded salaries at Band 7 compared to 37 % in physiological sciences. Consequently, more respondents from physiological sciences either remained on Band 6 compared with the other divisions or decreased to Band 5 (see Fig. 2).

### Interviewee characteristics

Thirteen volunteer early career scientists were interviewed, 4 from life sciences, 6 from physical sciences and 3 from physiological sciences (Table 1). All were at least two years post STP completion.

**Table 1:** Interviewee characteristics. Interviewee first job sector and salary at the time of the interview in 2018 are shown. Healthcare: non-NHS job sectors included universities (2 individuals) and private healthcare (1 individual)

Division	ID and specialty	Job sector	Salary
Life sciences	Clinical Biochemistry 1	Healthcare: NHS	Band 7
	Clinical Microbiology 1	Healthcare: Non-NHS	Band 7 equivalent
	Genetics 1	Healthcare: NHS	Band 7
	Reproductive Science 1	Healthcare: NHS	Band 8
Physical sciences	Clin. Pharmaceutical Science 1	Healthcare: NHS	Band 8
	Clin. Pharmaceutical Science 2	Healthcare: NHS	Band 7
	Medical Physics 1	Healthcare: NHS	Band 8b
	Medical Physics 2	Healthcare: Non-NHS	Band 7 equivalent
	Medical Physics 3	Healthcare: NHS	Band 7
	Medical Physics 4	Healthcare: NHS	Band 7
Physiological sciences	Audiology 1	Healthcare: NHS	Band 6
	Audiology 2	Healthcare: Non-NHS	Band 7 equivalent
	Audiology 3	Healthcare: NHS	Band 7

### Scientists’ early career choices and progression

**Fig. 3:**
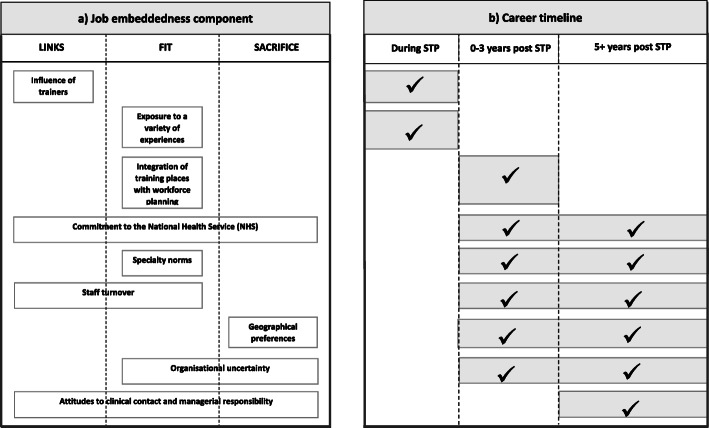
Influences on early career clinical scientists’ career choice and progression aligned to the three components within job embeddedness theory (**A**) and to a Career Timeline (**B**). The ticks show when the corresponding themes are influential relative to the career timeline

Interviewees described how their career progression was faster than they had anticipated and referred to the STP as a ‘gold standard’ qualification with a clear developmental pathway. Nine themes relating to influences on career choice and progression were identified and were grouped based on the stages they are influential (Fig. 3). During the STP, themes relating to influence of trainers and variety of training experiences were identified; whilst for early career scientists (0–3 years post STP), integration of training places with workforce planning, commitment to the NHS, specialty norms such as starting salaries, staff turnover, geographical preferences and organisational uncertainty were important. For longer term decision-making (5 + years post STP), attitudes to clinical contact and managerial responsibility were also influential.

### During STP

#### Influence of trainers

Enthusiastic, skilled and engaged trainers were a major influence on trainees’ career choice and progression, providing role models, mentoring and creating opportunities for learning:

… *my training officer… she was excellent. She has opened loads of doors for me… I feel like she is a role model to me. [Clinical Pharmaceutical Science 2]*

*I was quite lucky with my training manager who is a clinical scientist as well and she realised the extra skills that you would gain from doing the STP rather than doing an undergrad in Audiology. [Audiology 1]*

#### Exposure to a variety of experiences

The extent to which trainees were integrated into the work of their training department influenced the range of experience to which they were exposed. Interviewees viewed the breadth of skills that they developed in their workplace-based training as valuable for career choice and progression:

*I think it [the STP] has been really valuable in term of getting links and giving a broad appreciation for all different areas of genetics. I think if other training routes that they did before and are still somewhat existing, you end up with, you end up in a team doing one particular area and I think STP gives you a broad kind of overview [Genetics 1]*.

Opportunities to experience different areas of their specialty often sparked interest in a specific career trajectory:

*I had an idea of what other pharmaceutical scientists do in the NHS generally, but not about radio pharmacy. It’s kind of like a niche branch, so is nuclear medicine I guess, and then, as I was doing the STP, I realised that that was like what I enjoyed the most out of doing the four different specialities that you kind of studied [Clinical Pharmaceutical Science 1]*.

Differences between training departments led to differences in the range of experience and the level of competence achieved:

*I think I was quite lucky in my department because they were really keen for me to see everything and keep going with everything until I decide what not to do, or what to specialise in. [Audiology 1]*

*It depends a lot on your training department as to what you come out of the scheme with and the level you’re at really. [Medical Physics 1]*

### Early career (0–3 years post STP)

#### Integration of training places with workforce planning

The extent to which programme managers and host training departments aligned STP training places with workforce demand for particular specialties affected the availability of posts and therefore influenced career choices:

*…there were some people that were sort of guaranteed a job when they finished the STP programme or very likely to get a role within the department they were working. Whereas others knew that they wouldn’t necessarily have a job when they finished. [Clinical Pharmaceutical Science 2]*

*I don’t know about other specialisms, but I think in Audiology some departments take on a trainee with the knowledge that they’ve got that gap in what they need in the service. So they’re thinking of workforce planning. Whereas other departments take on two trainees, you know, every year, none of them ever get jobs. So I think again that’s something that really varies, depending on your host department, what your job prospects are afterwards. [Audiology 1]*

#### Commitment to the National Health Service (NHS)

Most interviewees saw their career progressing within the NHS and cited a range of reasons for this, including loyalty to the NHS, familiarity with NHS structures and a desire to maintain clinical contact with patients. Most interviewees felt they wanted to ‘give something back’ in return for the investment and effort in their training:

*I wanted to stay in this role … I do appreciate, share the ethos of the NHS: you know, to treat patients and to treat anyone that needs it. [Medical Physics 2]*

*I just believe in the NHS, I wouldn’t want to work for a private company. And also the NHS always supported me with my training so I certainly wouldn’t want to do all my training and then leave… I’ve done my training to be a clinical scientist in the NHS. [Clinical Biochemistry 1]*

Working in the NHS was an aspiration shared by interviewees with jobs in non-NHS sectors. For these scientists, contributing to the training of others gave them some satisfaction that they were ‘giving something back’.

*Part of me feels kind of cheeky that I’ve done the STP but I’m not working in the NHS, because obviously it funded it, so whether there should be a way to kind of make sure people get a job in the NHS. I feel like I’m giving back now because I’m doing more honorary work and I’m training the next generation of audiologists. [Audiology 2]*

*I would have absolutely loved to have stayed in the NHS but that wasn’t really an option that was feasible. [Clinical Microbiology 1]*

#### Specialty norms

Trainees were aware that starting grades and progression could differ between specialties, with interviewees from audiology reporting progressing more slowly than others:

*I expected to get a Band 7 when I finished [the STP] I guess because it’s the natural progression. [Clinical Pharmaceutical Science, 1]*

*Most of the people in my department are a Band 6, and once you start doing more specialist work, or take on some sort of management responsibilities, that’s when then you would go up a band [Audiology 1]*.

*It is completely specialism-dependent. But I suppose the idea with the STP was that everybody would be the same, which is great in theory but it’s completely not the same. [Clinical Microbiology 1]*

#### Staff turnover

Many interviewees reported relatively low staff turnover in their departments, which could limit their opportunities for progression:

*There’s very few 8a positions available. So sometimes it feels like it’s the case of waiting for someone to retire, or someone to move… so I think probably that part of my career progression would probably take longer than I would ideally like it to. [Genetics 1]*

*You don’t know when one [job] is going to come up. Previously it was a bit of a case of ‘dead man’s shoes’ and when, you know, someone retires that’s when posts appear but there are more and more posts appearing now*. *[Medical Physics 1]*

*Well yeah I guess because, you know, managers tend to stay for a long time before they retire. So I never thought there would have been an opportunity. [Reproductive Science 1]*

Some interviewees ascribed their progression to being in the ‘right place at the right time’:

*…it was just my luck that someone left. So we had a member of staff leave, she found another job … which meant there was a vacant position and my Head of Department decided that she thought there was, a sort of research gap and so she designed a Clinical Scientist role which, I applied for. [Clinical Pharmaceutical Science 2]*

#### Geographical preferences

Interviewees’ career choices were also influenced by where they preferred to live.

*…I wanted to stay in [City] and that [career progression] was potentially quite unlikely because if a job comes up you essentially have to go for it or risk not finding anything.” [Medical Physics 1]*.

Their personal situation, particularly their family commitments, affected their willingness to relocate:

*…all of my family are local, and so in terms of childcare it is much more convenient being here… I don’t know if much further down the line if I would move for a consultant position, but that is probably something I’d have to think about at the time. [Clinical Biochemistry 1]*

*… By that stage [end of STP] you’re mid-20 s minimum or older. If you’ve already got a life, and a job, and a house, well not necessarily a job, but a partner, a family, you’re not going to just up and relocate. [Audiology 2]*

#### Organisational uncertainty

Interviewees working in the NHS were realistic about possible changes to service delivery and working practices in their Trusts; and appreciated that these could affect their progression:

*You can’t predict what anything’s going to be like …[and] … how the NHS is going to be, because that’s affected by outside forces, like who’s health secretary and which government is in power.[Clinical Biochemistry 1]*.

*I still do have the dream of progressing on in the NHS, but in the current climate…the labs all becoming more and more privatised, I’ve kind of got to be realistic about the chances of that, and I think you know they’re pretty slim. [Clinical Microbiology 1]*

### Longer term (5 + years post STP)

With the exception of integration of training places with workforce planning, all factors affecting early career choice continued to be influential 5 + years post STP completion. In addition, attitudes to clinical contact and managerial responsibility became a factor.

#### Attitudes to clinical contact and managerial responsibility

Some interviewees were interested in taking on more managerial responsibilities and were confident that they could rise to the challenge. Others preferred to maintain a clinical focus, recognising that this might affect their career progression:

*I want to run my own department. I don’t want to stay stagnant… I keep on wanting to develop myself and also, I really enjoy working with people and I think that I do quite well in a managerial role. [Medical Physics 4]*

*I would like to carry on in my role and grow in experience here. I’m already in a development senior role which I’d like to continue. I don’t necessarily think I’d like to go higher because I think you start to lose the clinical contact…I think I’d like to stay clinical, but it’s nice to try and influence things and shape policies and processes and things. [Audiology 3]*

## Discussion

Most UK Scientist Training Programme (STP) completers who qualified between 2014 and 2019 obtained a first post in the UK National Health Service (NHS) and achieved the anticipated salary (AfC Band 7). Some variation between scientific divisions was apparent: life scientists’ first job sector was less likely than other divisions to be the NHS; and physiological scientists were less likely to achieve the expected rise in salary.

Interview responses indicated that experiences during training influenced career choice and progression. Exposure to new specialty areas during training could spark an interest that altered an individual’s career path; and enthusiastic, skilled and engaged training officers were a major influence on trainees’ thinking about their careers, providing role models, mentoring and creating opportunities for learning.

The extent to which STP training places aligned with demand for qualified scientists influenced career choice and progression on immediate qualification. Specialty norms, staff turnover, organisational uncertainty and personal factors such as preference for geographical location influenced them in both the short (0–3 years) and longer term (5 + years). Many interviewees reported a strong commitment to an ethos of public service, patient care and a desire to ‘give something back’ in return for their training and opportunities. This could explain why a high proportion of completers entered first employment in the National Health Service. In the future, some could foresee that these priorities would influence their decisions about whether or not to apply for a management and leadership position that would take them away from clinical work.

Mitchell’s job embeddedness theory offers a framework for understanding why ‘embedded’ individuals are more likely to remain in an organisation [11, 12]. The theory suggests that factors affecting retention can be grouped as ‘links’ (an individual’s informal and formal connections with people in their organisation), ‘fit’ (how well an individual’s personal values, goals and plans align with the organisational culture) and ‘sacrifice’ (material or psychological benefits that may be lost when an individual leaves). Our findings suggest that this theory also has utility for understanding the related concepts of career choice and progression. Influences on career choice identified by interviewees aligned well with job embeddedness components, particularly that of ‘fit’ (see Fig. 3A). In contrast, although Bronfenbrenner’s theory of human development seems comprehensive, as it considers the entire ‘ecological’ system surrounding the individual, we found it difficult to assign the influences identified in our study to particular systems within this framework.

Salary and opportunities for career progression varied across scientific specialities: trainees from the physiological sciences formed a substantial proportion of the 31 % of STP completers who did not achieve the expected salary on qualification; and some interviewees, particularly those from audiology, reported lower starting salaries and slower career progression than their peers in other specialties. This suggests that clinical science is a diverse group that is still influenced by historical speciality norms, despite recent initiatives to standardise training and career opportunities for healthcare scientists [22]. While such differences are difficult for managers and educators to influence, our findings suggest that attention to variations between specialties is important if career opportunities for all clinical scientists are to be enhanced.

Although many interviewees expressed a desire to work in the NHS, some felt that this was not an option open to them. This may reflect competing personal priorities, such as preference for a geographical location with limited availability of NHS posts. Organisational factors, including governmental priorities for outsourcing scientific services [23] and the extent to which STP training places reflect future workforce demand may also affect an individual’s ability to realise this ambition.

Influences on the career choices and progression of clinical scientists are similar to those identified for doctors. Experiences during training and exposure and role modelling within the specialty influence doctors’ choices [24–26], as does perceived loyalty to the UK National Health Service (4). For doctors, organisational uncertainty in the specialty, geography, job opportunities and staff shortages are also influential [4, 9, 26–28]. Like those affecting clinical scientists, factors affecting doctors’ career choices change over time (see Fig. 3B) [26, 28].

Our study suggests a need to enhance integration of clinical scientist training posts with NHS workforce planning. This complex task will require understanding of the perspectives and priorities of organisational stakeholders, as well as those of individual scientists. The parallels that we have observed between the clinical sciences and medicine suggest that mechanisms for ensuring medical training-workforce integration may be transferable to the clinical sciences; and leaders at the National School for Healthcare Sciences advocate the introduction of Scientific Directors at Trust Board level, akin to those already in place for medicine, to provide similar leadership and profile.. Investigation of all these aspects would be an appropriate follow up to our study.

Our study has a number of limitations. Our focus on interviewing scientists who had been in practice for at least 2–3 years meant that we were unable to obtain information from the small number of scientists in the then relatively new bioinformatics division. Although our sample of 13 interviewees included at least one scientist from each of the other three divisions, it was not possible to sample all scientific specialties. Our study is a snapshot of choices and progression at a particular career stage, and we acknowledge that such choices may change as careers develop over the longer term. Although the concept of job embeddedness has been questioned [29], it was particularly valuable to the qualitative aspect of this study; aligning our themes with ‘links, fit and sacrifice’ has helped to identify types of intervention that are appropriate to support career choice, and hence enhance retention. Fewer factors aligned with the job embeddedness components of links and sacrifice: this is not surprising, given our focus on career choice rather than reasons for leaving.

Our findings are encouraging for educators and workforce managers working to improve retention of skilled and motivated clinical scientists. They suggest that attention to variation in training experience, the quality of trainee mentoring and supervision and better integration of training places with likely workforce demand will pay dividends, as will maximising the ‘fit’ between individuals and their organisation. However, variation between specialties will need to be considered when developing interventions.

## Conclusions

Training experiences, personal values, specialty norms and organisational factors all influence UK clinical scientists’ early career choices and progression. These factors align with concepts within job embeddedness theory, particularly that of ‘fit’. Job embeddedness provides a useful lens through which to explore career choice and progression; and suggests types of intervention that can enhance the careers of this essential group. Interventions need to take account of variations between different scientific specialties.

## Supplementary information

**Additional file 1**

## Data Availability

The datasets generated and analysed during the current study are not publicly available due to the risk of identification of individual participants from combinations of data items. Datasets are available from the corresponding author on reasonable request.

## Abbreviations

AfC: Agenda for Change
NHS: National Health Service
STP: Scientist Training Programme
