# Supplementary material for: Clinical scientists’ early career choices and progression: an exploratory mixed methods study
Source: BMC Health Serv Res. 2021 Oct 6;21:1059. doi: 10.1186/s12913-021-07064-1 (PMC8494160; doi:10.1186/s12913-021-07064-1)
Supplement: Supplementary file 1 — Additional file 1 [file 12913_2021_7064_MOESM1_ESM.docx]

**Interview Schedule (telephone interview)**

Hi ….

This is __________ calling from the University of Birmingham with regards to the study of STP alumni career destinations. Can you hear me okay?

Thank you again for agreeing to participate in this study. There’s a few things I need to run through before we commence with the interview.

So…This research aims to investigate the career destinations of STP graduates. As a graduate, this study is keen to explore your experiences on the programme and how this may have informed your career choices.

I hope to make this conversation open and informal, so please share any experiences you think may be relevant.

I would like to remind you that, participation in this interview is completely voluntary, with your information being entirely confidential. Anything that is reported will remain anonymous; only non-identifiable information will be shared with the National School of Healthcare Science. A copy of your transcript will be available to you on request.

You have the right to withdraw at any point during the interview, and you do not have to answer all of the questions, this will not affect you in any way. The purpose of this interview is simply to gain a comprehensive understanding of your experiences on the STP and your career aspirations and destinations.

- Before we begin, do you have any further questions regarding this research?
- Please do not hesitate to ask any questions throughout the course of the interview.

**Interview topics and indicative questions**

So firstly…

**About you**

1. Can you tell me a little about yourself?

You may want to talk about yourself and what you did prior to the STP so what your degree was in, where you studied and also about the STP and…

- - *What specialty did you train in?*
  - *Where did you complete your training?*
  - *When did you graduate?*

**About your career**

1. Would you be able to tell us about your employment since you completed the STP?
   - *Are you still in the same job as you were after graduating?*
2. Could you tell us about your current role and your duties and responsibilities
3. Is this where you thought you would be when starting the STP?
   - If not, in what way is it different? Why do you think these differences have come about?

**About the Programme**

1. What attracted you to the STP?
2. Did you get the preferred specialty and location that you applied for?
3. What were you hoping to gain from the Programme?
4. What particular skills did you develop that were useful to your career?
5. How valuable do you think the STP was to your career?
6. What aspects of the programme helped you to progress in your career?
7. What aspects of the programme hindered your career progression?
8. Did you have any opportunities to form networks that may be useful throughout your career?
9. How well do you think the programme is valued by employers whilst on the programme?
   - (*Here the term employers is defined as the organisation who employed you throughout the three year training contract on the STP)*

**Graduation from the programme**

1. Did you apply for professional registration with the HCPC upon completion of the programme? If not, why (not)?
2. How easy was it to find a job after graduating from the programme?
3. *What were your motivations for **staying/ leaving** the NHS?
4. How much career support did you receive prior to graduation?
5. Being an STP graduate, how well do you think the programme is valued by employers?
6. If you could go back, would you undertake the programme again?
7. Would you recommend the STP programme to a friend? If so, why (not)?

**Role as a clinical/healthcare scientist**

1. Are you in the position you wanted to be in when you first set out?
2. How do you see your career progressing from here? Where would you like to be in 5-10 years?
3. Is there anything else you would like to share about your career progression?

**Questions to ask if interviewee is employed in the private sector*

**That concludes the interview. Thank you for giving up your time to take part in this study.**

**Your transcript will be available for you to view for you to correct any misunderstandings. Please let us know if you wish to view this.**

**You are able to withdraw from the study and you may do so before Friday 14^th^ September, which is when data analysis will begin.**

**If you have any further questions or queries, please contact me via email or telephone. My contact details can be found on the Participant Information Sheet.**

Thank you once again for participating in this study.
